# C‐Terminal Tail Elongation Adds a New Dimension to the Tubulin Code

**DOI:** 10.1002/cm.70088

**Published:** 2026-01-08

**Authors:** Jana Campbell, Cyril Barinka

**Affiliations:** ^1^ Institute of Biotechnology of the Czech Academy of Sciences, BIOCEV Vestec Czech Republic; ^2^ Department of Biochemistry, Faculty of Science Charles University Prague Czech Republic

## Abstract

Tubulin C‐terminal tails undergo diverse post‐translational modifications that regulate microtubule interactions with motors and severing enzymes. TTLL11, a member of the tubulin tyrosine ligase‐like (TTLL) family, uniquely catalyzes glutamate addition to the terminal α‐carboxyl group of both α‐ and β‐primary tubulin tails. This linear C‐terminal glutamylation enables the rescue of truncated tubulin variants and may support re‐entry into the modification cycle. TTLL11 substrate specificity is determined by terminal residue identity rather than tubulin isotype. These findings expand the tubulin code and raise new questions about how linear and branched glutamylation are differentially recognized by microtubule‐associated proteins.

## 1 Post‐Translational Modifications of Tubulin C‐Tails

Microtubules are hollow, cylindrical polymers composed of α‐ and β‐tubulin dimers (Mohri 1968). Each tubulin subunit consists of a well‐structured globular core and an intrinsically disordered C‐terminal tail (C‐tail) that extends outward from the microtubule surface (Amos 2004). These C‐tails are rich in glutamate residues, and the γ‐carboxyl groups of these glutamates serve as key sites for post‐translational modifications (PTMs), particularly glutamylation and glycylation (Roll‐Mecak 2020). Both are classified as polymodifications, meaning they can involve the sequential addition of multiple glutamate or glycine residues to the tail. This enables the generation of a gradient of signal that fine‐tunes microtubule behavior, such as interactions with motors and microtubule‐associated proteins (MAPs) (McKenna et al. 2023). These modifications are catalyzed by members of the tubulin tyrosine ligase‐like (TTLL) enzyme family, each of which displays unique substrate specificity and either chain‐branching or chain‐elongation properties (initiates and elongates, Figure 1A; Janke et al. 2005).

**FIGURE 1:**
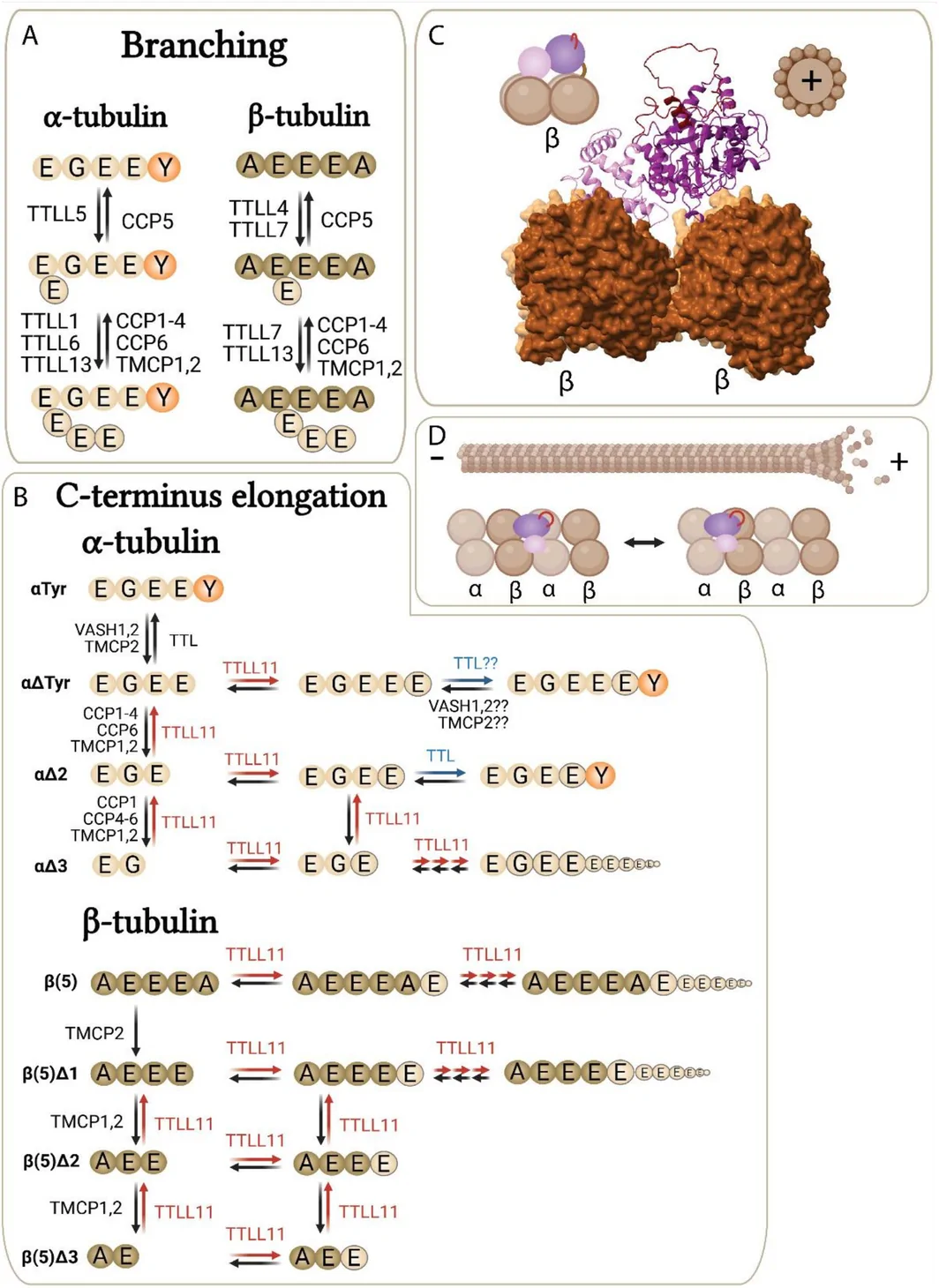
Tubulin tail PTMs and TTLL11 binding. (A) A traditional view of polyglutamylation—branching from glutamate sidechain on either α‐ or β‐tubulin. (B) Modifications of the C‐terminus with highlighted reactions catalyzed by TTLL11 (red). (C) TTLL11/microtubule complex, TTLL11 microtubule‐binding helix bundle (MT‐BHB, pink) binds one protofilament while the catalytic domain in placed over the adjacent protofilament (scheme and structure model in cartoon) facing the plus end of the microtubule. (D) TTLL11 can bind either of α/β and β/α grooves.

## 2 A Novel C‐Terminal Modification by TTLL11

Among the TTLL enzymes, TTLL11 exhibits a distinct catalytic behavior. Unlike other family members that modify internal γ‐carboxyl groups of glutamate residues and/or elongate the newly formed branch via α‐carboxyls, TTLL11 catalyzes the attachment of a glutamate residue directly to the C‐terminal α‐carboxyl group of the primary tubulin tail. This reaction introduces a previously unrecognized type of PTM, expanding the diversity of the tubulin code and introducing a new axis of regulatory complexity (Figure 1B). This activity represents a fundamental deviation from canonical glutamylation mechanisms and is particularly significant in the context of tubulin tail truncation and the modification cycle. In addition to TTLL11, TTLL6 also seems to possess the α‐tubulin C‐tail elongation capacity (Campbell et al. 2025; Mahalingan et al. 2024).

## 3 C‐Terminal Processing and Reversible PTM Recycling of α‐Tubulin

The C‐termini of both α‐ and β‐tubulin subunits are also subject to regulated proteolytic processing (Aillaud et al. 2017; Landskron et al. 2022; Nicot et al. 2023; Nieuwenhuis et al. 2017). Most α‐tubulin isoforms terminate in the sequence …GEEY, where the terminal tyrosine can be removed by vasohibin (VASH1/2) or TMCP1, and subsequently re‐added by tubulin tyrosine ligase (TTL), forming the well‐characterized tyrosination/detyrosination cycle (…GEEY ⇄ …GEE) (Aillaud et al. 2017; Nieuwenhuis et al. 2017). Beyond this reversible modification, further proteolytic cleavage by cytosolic carboxypeptidases (CCPs; Berezniuk et al. 2012) can remove one or two glutamate residues preceding the tyrosine, yielding truncated αΔ2 and αΔ3 forms, respectively. These truncated variants were historically considered irreversible “dead‐end” products—unable to participate in re‐tyrosination and presumed functionally inert in the PTM cycle (Aillaud et al. 2016; Paturle‐Lafanechere et al. 1994). However, recent work has demonstrated that TTLL11 can restore glutamates to the truncated C‐termini of αΔ2 and αΔ3 tubulins, effectively reinitiating them into the modification cycle (Figure 1B) (Campbell et al. 2025). This reveals an unexpected layer of plasticity in microtubule regulation and highlights the role of TTLL11 as a functional “rescue enzyme” capable of extending the lifespan and modification potential of otherwise inactivated tubulin variants.

Beyond rescuing truncated variants, TTLL11‐mediated elongation could also permit *neo‐tyrosination* of the extended C‐tail. In this scenario, added glutamates create a longer terminal chain that may serve as a new substrate for TTL, further expanding the tubulin code (Figure 1B). However, whether TLL can act on the elongated C‐tail added by TTLL11 is not known and warrants further study.

## 4 β‐Tubulin Tail Diversity and TTLL11 Selectivity Against C‐Terminal Residues

β‐Tubulin isoforms exhibit greater sequence variability at their C‐termini than α‐tubulin, yet they consistently possess glutamate‐rich tails that typically end in alanine. These tails are also susceptible to proteolytic truncation by the TMCP family (Nicot et al. 2023).

Importantly, TTLL11 catalyzes C‐terminal glutamylation on both truncated and full‐length β‐tubulin tails (Campbell et al. 2025). Variants ending in glutamate are the most efficiently glutamylated, followed by those ending in small, uncharged residues like alanine or glycine. In contrast, α‐tubulin tails terminating in bulky, aromatic residues—such as tyrosine—are not glutamylated at their C‐termini by TTLL11 (Campbell et al. 2025). This suggests that TTLL11 discriminates substrate tubulin variants based on C‐tail sequence, thereby adding selective remodeling potential to the tubulin code.

A similar substrate preference was observed for another TTLL11 target, Dishevelled‐3 (Dvl3). Mutagenesis of its C‐terminus revealed a clear preference for glutamate residues over native methionine, and further analysis showed that the presence of negatively charged amino acids within the tail is required for TTLL11 recognition (Kravec et al. 2024). All tubulin C‐termini naturally contain multiple glutamate residues that confer this negative charge, which likely underlies their compatibility with TTLL11.

## 5 A Unique Binding Mode Underlying TTLL11 Specificity

This unusual substrate specificity is unique within the TTLL family. While other TTLL enzymes exhibit isotype selectivity—TTLL6 preferentially glutamylates α‐tubulin (Aillaud et al. 2016; Mahalingan et al. 2020), and TTLL7 targets β‐tubulin (Garnham et al. 2015)—TTLL11 modifies both α‐ and β‐tubulin, with substrate preference dictated by C‐tail sequence composition, particularly the terminal residue (Campbell et al. 2025). This preference is most likely enabled by a distinctive microtubule‐binding mechanism that differs substantially from those employed by other TTLLs.

Canonical TTLLs typically bind and modify the same protofilament using a tri‐ or quadripartite mechanism involving the catalytic domain, microtubule‐binding domain, and microtubule‐binding helices (Mahalingan et al. 2024; Garnham et al. 2015). In contrast, TTLL11 utilizes a bipartite binding strategy exploiting a novel microtubule‐binding helix bundle (MT‐BHB), which lacks sequence similarity to known TTLL elements, together with a catalytic domain that engages with the C‐terminal tail. Each interaction is with the adjacent protofilament (Figure 1C). Structural analysis reveals that the MT‐BHB anchors within the α/β‐tubulin groove of a single protofilament, interacting symmetrically with either α–β or β–α interfaces due to the conserved nature of the groove contact residues (Campbell et al. 2025). Consequently, TTLL11 is most likely not constrained by tubulin isotype at the level of binding affinity, and the determinant of substrate preference is reduced to the C‐tail sequence itself (Figure 1D).

## 6 Tubulin C‐Terminus Elongation Suggests Unexplored Layers of Microtubule Control

TTLL11 introduces a unique layer to the tubulin code by catalyzing C‐terminal glutamylation, rescuing truncated tubulin C‐tails, and modifying both α‐ and β‐tubulin independent of isotype, at least in vitro. At the same time, high glutamate abundance in tubulin C‐termini complicates studies of tubulin C‐terminal PTMs in vivo. Distinguishing between native residues and those added during glutamylation requires the use of isotopically labelled glutamates—feasible in in vitro systems but nearly impossible in vivo. This technical limitation likely explains why C‐terminal elongation by TTLL11 has been characterized only recently.

There is still a lot to unveil regarding TTLL11 functions in the organism and the co‐play of it with all the other MT modifying enzymes in different cell types and organelles. Microtubule‐associated proteins (MAPs) such as spastin, katanin, dynein, and kinesins are known to respond to specific glutamylation patterns, often in an isotype‐dependent manner (Genova et al. 2023; Lacroix et al. 2010; Sirajuddin et al. 2014; Szczesna et al. 2022). So far, these interactions have been studied primarily in the context of *branched* side‐chain glutamylation. The discovery of TTLL11‐mediated linear C‐terminal elongation raises compelling questions regarding whether and how MAPs can discriminate between branched and linear forms of glutamylation, and what consequences this might have for microtubule severing, motor activity, and signaling.

Taken together, TTLL11 not only broadens the enzymatic capabilities within the TTLL family but also emerges as a potential regulator of microtubule function, with the capacity to integrate tubulin truncation states into a dynamic post‐translational modification network.

## Funding

This work was supported by Czech Academy of Sciences, 86652036; Grantová Agentura, Univerzita Karlova, 284023; Grantová Agentura České Republiky, GA23‐07149S.

## Conflicts of Interest

The authors declare no conflicts of interest.

## Data Availability

Data sharing not applicable to this article as no datasets were generated or analyzed during the current study.
